# Eye Care-Seeking Behavior Among Staff of the University of Benin, Benin City, Nigeria

**DOI:** 10.7759/cureus.83286

**Published:** 2025-05-01

**Authors:** George N Atuanya, Oghenetekevwe M Oke, Babatunde I Bale, Musa Mutali

**Affiliations:** 1 Optometry, University of Benin, Benin City, NGA; 2 Ophthalmology, Africa Eye Laser Centre, Benin City, NGA

**Keywords:** eye care seeking, eye health awareness, health seeking behavior, nigeria eye care, university staff health

## Abstract

Background: Eye care-seeking behavior plays an important role in addressing preventable vision loss and maintaining optimal eye health, yet significant gaps exist in the utilization of eye care services, particularly in developing countries like Nigeria. This study investigated the factors influencing eye care-seeking behavior among staff of the University of Benin, Benin City, Nigeria.

Methods: A cross-sectional descriptive study was conducted using a stratified random sampling technique among staff members from all faculties of the University of Benin. A structured questionnaire was used to obtain information on sociodemographic factors influencing their eye care-seeking behavior, awareness of available eye care services, and barriers to accessing care. The analysis was carried out with IBM SPSS Statistics for Windows, Version 25.0 (Released 2017; IBM Corp., Armonk, New York, United States) using descriptive statistics and chi-square tests with an alpha level at 0.05.

Results: Of the 371 participants, 213 (57.40%) were female and 158 (42.60%) were male. The mean age was 43.21 ± 11.24 years (range 20-69). Most of the participants used the National Health Insurance Scheme as their health finance plan coverage (56.30%), which was significantly associated with their eye health seeking behavior (*p* < 0.05). Gender was significantly associated with having an eye examination in the last 1-3 years (*p* = 0.042), seeking care at private eye clinics (*p* = 0.02), consulting an optometrist (p < 0.001), and visiting an eye clinic for their most recent eye problem (*p* < 0.001). Most participants recognized the importance of regular check-ups (78.40%) and are familiar with the different types of eye care services (n=209; 52.50%), however, 39.1% were unaware of the recommended examination frequency. The most common barriers were lack of awareness about available eye care services (73.3%), work-related commitments (73.1%), and fear of diagnosis or treatment (70.7%).

Conclusion: This study showed that while awareness of eye care is relatively high among staff at the University of Benin, various barriers prevent the effective utilization of the services, especially a lack of awareness about available eye care services. Therefore, targeted interventions are recommended to improve awareness and access to eye care services among university staff.

## Introduction

Globally, the World Health Organization (WHO) estimates that at least 2.2 billion people live with vision impairment or blindness, of which over one billion cases could have been prevented or have yet to be addressed [1]. The ability to maintain optimal eye health is essential not only for an individual’s well-being but also for societal economic productivity. Visual impairment and blindness can significantly affect a person’s quality of life, reducing their capacity to perform daily activities [2]. Despite these consequences, many individuals often neglect regular eye examinations and delay seeking professional care until vision problems become severe [3]. This global burden of eye disease underscores the critical need for effective eye care services and the promotion of eye health-seeking behavior among populations.

In Nigeria, access to eye care services remains a significant public health challenge. The prevalence of eye disease conditions such as cataract, glaucoma, refractive errors, and diabetic retinopathy is on the rise, exacerbated by an ageing population and increasing rates of non-communicable diseases like diabetes and hypertension [4]. Limited access to appropriate eye care services is one of the drawbacks to reducing blindness in developing countries like Nigeria [5]. WHO reported that the utilization of eye care services globally was 18% in 2012 [6]. Similarly, it has been reported that utilization of existing eye care infrastructure in Nigerian communities is as low as 25% compared to the optimum target utilization set at 90% [7]. Consequently, people who live in communities with inadequate or inaccessible eye care facilities tend to seek other alternatives to eye care services [8]. 

Eye care-seeking behavior is a critical aspect of public health, particularly in developing countries where access to healthcare services can be limited [9]. Eye care-seeking behavior encompasses the actions and decisions individuals make regarding the maintenance of their eye health, including routine eye examinations, the use of corrective lenses, and seeking medical attention for eye problems [10]. Key influencing factors include socioeconomic status, education, cultural beliefs, awareness, service accessibility, and perceived severity of symptoms [11]. Geographic disparities, especially between urban and rural areas, further exacerbate inequities in access [12].

Previous studies on eye care-seeking behavior in Nigeria have predominantly focused on the general population. For example, Atuanya and Chilaka conducted a cross-sectional study among commercial drivers in Benin City, Nigeria, to assess their knowledge and attitudes toward utilizing eye care services [13]. The study showed that poor utilization of eye care services persisted within the population despite good knowledge. Similarly, Arinze et al. found that only 42.4% of rural residents in Nigeria had ever visited an eye clinic, citing lack of awareness (31.8%), cost (18.0%), and perceived treatment futility (15.9%) as primary barriers [14]. Conversely, those with health insurance, a family history of eye conditions, or noticeable vision changes were more likely to seek care.

However, evidence gaps persist regarding eye care-seeking behaviors among university staff, a population reliant on good vision for professional tasks [15]. Therefore, this present study aimed to bridge the gap. This study examined the association between sociodemographic factors and eye care-seeking behavior. It also determined awareness of eye care delivery and assessed whether specific barriers influenced differences in seeking eye care among staff of the University of Benin.

## Materials and methods

Study design, setting, and duration

This cross-sectional study evaluated eye health-seeking behaviors and barriers to accessing care among staff at the University of Benin, Benin City, Edo State, Nigeria. The university is a federal institution with over 6,000 academic and non-academic staff (during the 2019/2020 academic session), serving as a representative setting for assessing occupational eye care practices. Data collection occurred over five months (from October 2024 to February 2025) to ensure adequate time for participant recruitment and questionnaire administration. This study was guided by Andersen’s Behavioral Model [11], which categorizes factors influencing health-seeking behavior into predisposing (e.g., gender, age), enabling (e.g., health insurance), and need factors (e.g., ocular symptoms). This framework informed the selection of socio-demographic and barrier variables analyzed in this study.

Inclusion and exclusion criteria

Eligible participants were full-time or part-time University of Benin staff aged 18 years or older who provided written informed consent. The age criterion ensured compliance with Nigeria’s legal definition of adulthood and ethical standards for autonomous decision-making. Exclusion criteria comprised non-staff members (e.g., temporary contractors, students), individuals below 18, and those who declined consent.

Sampling technique

A stratified random sampling technique was employed to enhance representativeness. The population was divided into strata: academic staff (N_1_ = 2,516) and non-academic staff (N_2_ = 3,773), based on the University of Benin’s most recent internal report [16] for the 2019/2020 session. Participants were randomly selected using a computer-generated random number table to ensure unbiased sampling. The sample size was determined using the Charan & Biswas formula [17], $n = \frac{n_0}{1 + \frac{n_0 - 1}{N}}$ , with an expected proportion (*P*) of 63.6% derived from a similar previous study by Abdul-Kabir et al. [15]. With a 95% confidence interval (CI) (*Z* = 1.96), and a 5% margin of error (*d* = 0.05). Thus, the calculated sample size (*n*) was 337. However, to account for a 10% attrition rate, the final sample size was adjusted to 371 participants.

Data collection

Data were collected using a structured questionnaire adapted from a previous study by Abdul-Kabir et al. [15]. The questionnaire was reviewed and validated by two optometry experts to ensure relevance and clarity. Before the main study, a pilot test was conducted with 20 staff members to refine the questionnaire and identify any ambiguities. The final questionnaire comprised four sections: (1) sociodemographic information, (2) eye health-seeking behaviors, (3) barriers to eye care-seeking behavior, and (4) awareness of eye care delivery.

Trained research assistants administered the questionnaire. Written informed consent was obtained from all respondents before participation. To maintain confidentiality, no personally identifiable information was collected, and each questionnaire was assigned a unique code. 

The sociodemographic section captured details such as age, gender, marital status, highest educational qualification, job rank, and health financing plan. The eye health-seeking behavior section assessed the frequency of eye examinations, history of ocular symptoms, and utilization of optometry services. The barriers section evaluated challenges such as cost, distance, lack of awareness, and time constraints using a five-point Likert scale. The awareness section assessed awareness level about the importance of eye check-ups, available eye care services, and frequency of eye examinations, also using a five-point Likert scale.

Statistical analysis

Following data collection, entries were subjected to statistical analysis using IBM SPSS Statistics for Windows, Version 25.0 (Released 2017; IBM Corp., Armonk, New York, United States). Descriptive statistics such as means, standard deviations, and frequencies were used to summarize the sociodemographic characteristics. Chi-square tests were done to examine associations between categorical variables, such as gender in relation to eye care-seeking behavior, with statistical significance set at p < 0.05.

## Results

A total of 371 respondents participated in this study, of which 213 (57.40%) were female and 158 (42.60%) were male, with the majority falling within the 30-39 age range (38.80%). The age range of participants was 20-69 years, with a mean age of 43.21 ± 11.24 years. Most of the participants were married (69.80%), held tertiary education (68.70%), and were primarily senior academic staff (35.60%). Regarding health finance coverage, 56.30% were enrolled in the National Health Insurance Scheme (NHIS). This is presented in Table 1.

**Table 1:** Sociodemographics of the participants (N=371) NHIS: National Health Insurance Scheme

Variables		Frequency	Percentage
Sex	Female	213	57.40
	Male	158	42.60
Age group (years)	20-29	27	7.30
	30-39	144	38.80
	40-49	86	23.20
	50-59	78	21.00
	60-69	36	9.70
Marital Status	Divorced/Separated	7	1.90
	Married	259	69.80
	Single	83	22.40
	Widow/Widower	22	5.90
Educational Level	Senior High School	17	4.60
	Technical	67	18.10
	Tertiary	255	68.70
	Vocational	32	8.60
Job Rank	Junior Academia	80	21.60
	Junior non-Teaching	45	12.10
	Senior non-Teaching	114	30.70
	Senior Academic	132	35.60
Health Finance Plan	NHIS	209	56.30
	Others	8	2.20
	Out of Pocket	154	41.50

Table 2 showed that the age group of 30-39 years had the largest proportion (30.9%; n = 60) of participants with recent eye exams (within one to three years), while older adults aged 50-59 years mostly visited private clinics (24.8%; n = 34). The 30-39 age group also sought optometric consultation most frequently (26.4%; n = 42) and represented the majority visiting eye clinics for eye-related issues (27.3%; n = 48). Statistical analysis indicated non-significant age group differences in eye health-seeking behaviors across all categories (p > 0.05), including the timing of the last eye examination (p = 0.070), private eye care service (p = 0.800), choice of eye care provider (p = 0.360), and actions taken during their last eye problem (p = 1.240).

**Table 2:** Correlation between age and eye care-seeking behavior

Parameters		Age Group (years)											Pearson χ²	Df	P-value
		20-29		30-39		40-49		50-59		60-69		Total			
		n	%	n	%	n	%	n	%	n	%	N			
Last eye examination	1-3 years	21	10.8%	60	30.9%	39	20.1%	47	24.2%	27	13.9%	194	106.225	16	0.070
	I do not remember	3	8.1%	17	45.9%	10	27.0%	7	18.9%	0	0.0%	37			
	More than 3 years	0	0.0%	15	23.1%	17	26.2%	24	36.9%	9	13.8%	65			
	Never	3	4.0%	52	69.3%	20	26.7%	0	0.0%	0	0.0%	75			
Where do you usually go for eye care services?	Others	3	3.7%	55	67.1%	20	24.4%	4	4.9%	0	0.0%	82	147.299	16	0.360
	Private eye clinic	20	14.6%	28	20.4%	28	20.4%	34	24.8%	27	19.7%	137			
	Public hospital	0	0.0%	36	47.4%	9	11.8%	31	40.8%	0	0.0%	76			
	Traditional healer	0	0.0%	5	100.0%	0	0.0%	0	0.0%	0	0.0%	5			
	University of Benin	4	5.6%	20	28.2%	29	40.8%	9	12.7%	9	12.7%	71			
Distribution of eye care providers you have sought in the past	General practitioner	0	0.0%	7	46.7%	0	0.0%	8	53.3%	0	0.0%	15	109.000	20	0.800
	I do not remember	6	4.3%	73	52.9%	35	25.4%	15	10.9%	9	6.5%	138			
	Ophthalmic nurse	0	0.0%	9	81.8%	2	18.2%	0	0.0%	0	0.0%	11			
	Ophthalmologist	0	0.0%	7	25.0%	4	14.3%	17	60.7%	0	0.0%	28			
	Optician	0	0.0%	6	30.0%	9	45.0%	0	0.0%	5	25.0%	20			
	Optometrist	21	13.2%	42	26.4%	36	22.6%	38	23.9%	22	13.8%	159			
Action taking during last eye problem	Irrigation of eyes	0	0.0%	12	70.6%	4	23.5%	1	5.9%	0	0.0%	17	98.609	20	1.240
	Nothing	3	13.6%	12	54.5%	4	18.2%	3	13.6%	0	0.0%	22			
	Self-medication	4	5.1%	46	59.0%	19	24.4%	9	11.5%	0	0.0%	78			
	Visited a hospital	3	6.7%	18	40.0%	9	20.0%	15	33.3%	0	0.0%	45			
	Visited a pharmacy	0	0.0%	12	36.4%	15	45.5%	2	6.1%	4	12.1%	33			
	Visited an eye clinic	17	9.7%	44	25.0%	35	19.9%	48	27.3%	32	18.2%	176			

Female participants reported higher utilization rates for recent eye examinations within one to three years (62.4%, n = 121) compared to male participants (37.6%, n = 73), a finding supported by a significant association (p = 0.042). While both genders sought services at private eye clinics, female participants accounted for 49.6% (n = 68) of these visits versus male participants at 50.4% (n = 69), with a significant gender difference (p = 0.023). A higher proportion of female participants reported prior optometric consultations (39.91%, n = 85) compared to male participants (46.84%, n = 74), though this difference was statistically significant (p = 0.000). Visitation to eye clinics was more common among male participants (56.33%, n = 89) than female participants (40.85%, n = 87), and showed a significant association (p = 0.000). This is presented in Table 3.

**Table 3:** Correlation between gender and eye care-seeking behavior

Parameters		Gender					Pearson χ²	Df	P-value
		Female		Male		Total			
		n	%	n	%	N			
Last eye examination	1-3 years	121	62.4%	73	37.6%	194	9.928	4	0.042
	I do not remember	23	62.2%	14	37.8%	37			
	More than 3 years	27	41.5%	38	58.5%	65			
	Never	42	56.0%	33	44.0%	75			
Where do you usually go for eye care services?	Others	49	59.8%	33	40.2%	82	11.387	4	0.023
	Private eye clinic	68	49.6%	69	50.4%	137			
	Public hospital	44	57.9%	32	42.1%	76			
	Traditional healer	5	100.0%	0	0.0%	5			
	University of Benin	47	66.2%	24	33.8%	71			
Distribution of eye care providers you have sought in the past	General practitioner	13	86.7%	2	13.3%	15	29.184	5	0.000
	I do not remember	74	53.6%	64	46.4%	138			
	Ophthalmic nurse	2	18.2%	9	81.8%	11			
	Ophthalmologist	21	75.0%	7	25.0%	28			
	Optician	18	90.0%	2	10.0%	20			
	Optometrist	85	53.5%	74	46.5%	159			
Action taken during last eye problem	Irrigation of eyes	11	64.7%	6	35.3%	17	35.023	5	0.000
	Nothing	18	81.8%	4	18.2%	22			
	Self-medication	46	59.0%	32	41.0%	78			
	Visited a hospital	39	86.7%	6	13.3%	45			
	Visited a pharmacy	12	36.4%	21	63.6%	33			
	Visited an eye clinic	87	49.4%	89	50.6%	176			

Married participants comprised the majority across all categories, representing 72.2% (n = 140) of those with recent eye exams (within one to three years), 78.8% (n = 108) of participants that visited private eye clinics, 71.1% (n = 113) of those that sought optometric services, and 71.0% (n = 125) of respondents that visited an eye clinic for their last eye problems. Statistical analysis showed no significant association between marital status and eye health-seeking behaviors in any of these categories (p > 0.05), as detailed in Table 4.

**Table 4:** Correlation between marital status and eye care-seeking behavior

Parameters		Marital Status									Pearson χ²	Df	P-value
		Divorced/Separated		Married		Single		Widow/Widower		Total			
		n	%	n	%	n	%	n	%	N			
Last eye examination	1-3 years	2	1.0%	140	72.2%	35	18.0%	17	8.8%	194	53.654	12	0.200
	I do not remember	0	0.0%	32	86.5%	5	13.5%	0	0.0%	37			
	More than 3 years	2	3.1%	50	76.9%	8	12.3%	5	7.7%	65			
	Never	3	4.0%	37	49.3%	35	46.7%	0	0.0%	75			
Where do you usually go for eye care services?	Others	3	3.7%	50	61.0%	29	35.4%	0	0.0%	82	65.530	12	0.090
	Private eye clinic	4	2.9%	108	78.8%	19	13.9%	6	4.4%	137			
	Public hospital	0	0.0%	62	81.6%	8	10.5%	6	7.9%	76			
	Traditional healer	0	0.0%	0	0.0%	5	100.0%	0	0.0%	5			
	University of Benin	0	0.0%	39	54.9%	22	31.0%	10	14.1%	71			
Distribution of eye care providers you have sought in the past	General practitioner	0	0.0%	13	86.7%	0	0.0%	2	13.3%	15	80.407	15	0.210
	I do not remember	3	2.2%	80	58.0%	55	39.9%	0	0.0%	138			
	Ophthalmic nurse	0	0.0%	11	100.0%	0	0.0%	0	0.0%	11			
	Ophthalmologist	0	0.0%	22	78.6%	2	7.1%	4	14.3%	28			
	Optician	0	0.0%	20	100.0%	0	0.0%	0	0.0%	20			
	Optometrist	4	2.5%	113	71.1%	26	16.4%	16	10.1%	159			
Action taken during last eye problem	Irrigation of eyes	1	5.9%	11	64.7%	5	29.4%	0	0.0%	17	57.503	15	0.320
	Nothing	0	0.0%	14	63.6%	8	36.4%	0	0.0%	22			
	Self-medication	3	3.8%	43	55.1%	32	41.0%	0	0.0%	78			
	Visited a hospital	0	0.0%	40	88.9%	3	6.7%	2	4.4%	45			
	Visited a pharmacy	1	3.0%	26	78.8%	6	18.2%	0	0.0%	33			
	Visited an eye clinic	2	1.1%	125	71.0%	29	16.5%	20	11.4%	176			

Tertiary-educated participants comprised 85.6% (n = 166) of those with a recent eye exam within 1-3 years, with higher rates also observed for private eye clinic visits (75.9%; n = 104), optometric consultations (88.7%; n = 141), and eye clinic visitation (80.7%; n = 142) for their last eye problems. Statistical analysis showed no significant association between educational level and eye health-seeking behaviors across all categories (p > 0.05), as shown in Table 5.

**Table 5:** Correlation between educational level and eye care-seeking behavior

Parameters		Education Level									Pearson χ²	Df	P-value
		Senior High School		Technical		Tertiary		Vocational		Total			
		n	%	n	%	n	%	n	%	N			
Last eye examination	1-3 years	0	0.0%	20	10.3%	166	85.6%	8	4.1%	194	94.883	12	0.053
	I do not remember	1	2.7%	11	29.7%	17	45.9%	8	21.6%	37			
	More than 3 years	0	0.0%	17	26.2%	41	63.1%	7	10.8%	65			
	Never	16	21.3%	19	25.3%	31	41.3%	9	12.0%	75			
Where do you usually go for eye care services?	Others	16	19.5%	19	23.2%	34	41.5%	13	15.9%	82	91.921	12	0.230
	Private eye clinic	0	0.0%	22	16.1%	104	75.9%	11	8.0%	137			
	Public hospital	0	0.0%	17	22.4%	55	72.4%	4	5.3%	76			
	Traditional healer	0	0.0%	5	100.0%	0	0.0%	0	0.0%	5			
	University of Benin	1	1.4%	4	5.6%	62	87.3%	4	5.6%	71			
Distribution of eye care providers you have sought in the past	General practitioner	0	0.0%	0	0.0%	15	100.0%	0	0.0%	15	148.890	15	0.500
	I do not remember	17	12.3%	40	29.0%	59	42.8%	22	15.9%	138			
	Ophthalmic nurse	0	0.0%	9	81.8%	0	0.0%	2	18.2%	11			
	Ophthalmologist	0	0.0%	1	3.6%	27	96.4%	0	0.0%	28			
	Optician	0	0.0%	7	35.0%	13	65.0%	0	0.0%	20			
	Optometrist	0	0.0%	10	6.3%	141	88.7%	8	5.0%	159			
Action taken during last eye problem	Irrigation of eyes	0	0.0%	5	29.4%	12	70.6%	0	0.0%	17	96.019	15	0.780
	Nothing	0	0.0%	5	22.7%	14	63.6%	3	13.6%	22			
	Self-medication	16	20.5%	20	25.6%	31	39.7%	11	14.1%	78			
	Visited a hospital	1	2.2%	1	2.2%	39	86.7%	4	8.9%	45			
	Visited a pharmacy	0	0.0%	9	27.3%	17	51.5%	7	21.2%	33			
	Visited an eye clinic	0	0.0%	27	15.3%	142	80.7%	7	4.0%	176			

Senior academic staff constituted the largest group (37.6%; n = 73) that reported their last eye examination within one to three years, with a majority that visited private eye clinics (38.0%; n = 52) and sought optometric consultations (40.3%; n = 64), while senior non-teaching staff showed slightly higher rates of eye clinic visits (39.8%; n = 70). Statistical analysis showed no significant association between professional job rank within academia and eye health-seeking behaviors (p > 0.05), as shown in Table 6.

**Table 6:** Correlation between job rank and eye care-seeking behavior

Parameters		Job Rank									Pearson χ²	Df	P-value	
		Junior Academia		Junior Non-Teaching		Senior Academic		Senior Non-Teaching		Total				
		N	%	N	%	N	%	N	%	N				
Last eye examination	1-3 years	39	20.1%	16	8.2%	73	37.6%	66	34.0%	194	69.379	12		0.120
	I do not remember	2	5.4%	5	13.5%	16	43.2%	14	37.8%	37				
	More than 3 years	17	26.2%	0	0.0%	18	27.7%	30	46.2%	65				
	Never	22	29.3%	24	32.0%	7	9.3%	22	29.3%	75				
Where do you usually go for eye care services?	Others	22	26.8%	24	29.3%	14	17.1%	22	26.8%	82	46.613	12		0.610
	Private eye clinic	30	21.9%	10	7.3%	52	38.0%	45	32.8%	137				
	Public hospital	17	22.4%	4	5.3%	27	35.5%	28	36.8%	76				
	Traditional healer	0	0.0%	0	0.0%	0	0.0%	5	100.0%	5				
	University of Benin	11	15.5%	7	9.9%	21	29.6%	32	45.1%	71				
Distribution of eye care providers you have sought in the past	General practitioner	2	13.3%	0	0.0%	11	73.3%	2	13.3%	15	122.743	15		1.330
	I do not remember	35	25.4%	33	23.9%	20	14.5%	50	36.2%	138				
	Ophthalmic nurse	11	100.0%	0	0.0%	0	0.0%	0	0.0%	11				
	Ophthalmologist	8	28.6%	0	0.0%	15	53.6%	5	17.9%	28				
	Optician	0	0.0%	0	0.0%	4	20.0%	16	80.0%	20				
	Optometrist	24	15.1%	12	7.5%	64	40.3%	59	37.1%	159				
Action taking during last eye problem	Irrigation of eyes	1	5.9%	6	35.3%	3	17.6%	7	41.2%	17	81.024	15		2.060
	Nothing	3	13.6%	5	22.7%	3	13.6%	11	50.0%	22				
	Self-medication	30	38.5%	14	17.9%	12	15.4%	22	28.2%	78				
	Visited a hospital	15	33.3%	5	11.1%	22	48.9%	3	6.7%	45				
	Visited a pharmacy	4	12.1%	5	15.2%	5	15.2%	19	57.6%	33				
	Visited an eye clinic	27	15.3%	10	5.7%	69	39.2%	70	39.8%	176				

NHIS enrollees majorly utilized private clinics (72.3%; n = 99), reported recent eye examinations within one to three years (66.5%; n = 129), sought optometric consultations (62.9%; n = 100), and visited eye clinics (67.6%; n = 119). Health financing showed significant associations with eye healthcare-seeking behaviors, particularly in the last eye examinations (p = 0.020), private eye clinics visitation (p = 0.000), choice of eye care provider (p = 0.000), and actions taken during their most recent eye problem (p = 0.002). This is outlined in Table 7.

**Table 7:** Correlation between health finance plan and eye care-seeking behavior

Parameters		Health Finance Plan							Pearson χ²	Df	P-value
		NHIS		Others		Out of pocket		Total			
		n	%	n	%	n	%	N			
Last eye examination	1-3 years	129	66.5%	7	3.6%	58	29.9%	194	30.989	8	0.020
	I do not remember	21	56.8%	0	0.0%	16	43.2%	37			
	More than 3 years	29	44.6%	1	1.5%	35	53.8%	65			
	Never	30	40.0%	0	0.0%	45	60.0%	75			
Where do you usually go for eye care services?	Others	39	47.6%	0	0.0%	43	52.4%	82	49.224	8	0.000
	Private eye clinic	99	72.3%	2	1.5%	36	26.3%	137			
	Public hospital	26	34.2%	5	6.6%	45	59.2%	76			
	Traditional healer	0	0.0%	0	0.0%	5	100.0%	5			
	University of Benin	45	63.4%	1	1.4%	25	35.2%	71			
Distribution of eye care providers you have sought in the past	General practitioner	8	53.3%	0	0.0%	7	46.7%	15	41.253	10	0.000
	I do not remember	63	45.7%	5	3.6%	70	50.7%	138			
	Ophthalmic nurse	2	18.2%	0	0.0%	9	81.8%	11			
	Ophthalmologist	17	60.7%	0	0.0%	11	39.3%	28			
	Optician	19	95.0%	1	5.0%	0	0.0%	20			
	Optometrist	100	62.9%	2	1.3%	57	35.8%	159			
Action takeng during last eye problem	Irrigation of eyes	9	52.9%	0	0.0%	8	47.1%	17	28.251	10	0.002
	Nothing	12	54.5%	0	0.0%	10	45.5%	22			
	Self-medication	30	38.5%	1	1.3%	47	60.3%	78			
	Visited a hospital	22	48.9%	1	2.2%	22	48.9%	45			
	Visited a pharmacy	17	51.5%	0	0.0%	16	48.5%	33			
	Visited an eye clinic	119	67.6%	6	3.4%	51	29.0%	176			

Most of the participants were aware of the importance of regular eye check-ups for maintaining eye health (78.40%; n = 291) and were familiar with the different types of eye care services available (such as optometry and ophthalmology) (52.50%; n = 195). Most of the participants knew where to find information about eye care services in Benin City (72.50%; n = 269), were aware of common eye diseases and their symptoms (51.80%; n = 192), and understood the potential long-term impacts of untreated eye problems (70.40%; n = 261). However, the majority of the participants did not regularly receive information or updates about eye health from media, seminars, or health campaigns (57.50%; n = 213), and also did not know the recommended frequency for eye examinations based on their age and health condition (39.10%; n = 145). This is presented in Table 8.

**Table 8:** Awareness of eye care delivery among staff of the University of Benin

Awareness of eye care delivery	Strongly Agree	Agree	Neutral	Disagree	Strongly Disagree
I am aware of the importance of regular eye check-ups for maintaining eye health.	101 (27.20%)	190 (51.20%)	53 (14.30%)	27 (7.30%)	0 (0.00%)
I am familiar with the different types of eye care services available, e.g., optometry, ophthalmology.	68 (18.30%)	127 (34.20%)	86 (23.20%)	90 (24.30%)	0 (0.00%)
I know where to find information about eye care services in Benin City.	69 (18.60%)	200 (53.90%)	59 (15.90%)	43 (11.60%)	0 (0.00%)
I am aware of common eye diseases, e.g., cataract, glaucoma, etc., and their symptoms.	57 (15.40%)	135 (36.40%)	95 (25.60%)	70 (18.90%)	14 (3.70%)
I understand the potential long-term impacts of untreated eye problems.	69 (18.60%)	192 (51.80%)	61 (16.40%)	45 (12.10%)	4 (1.10%)
I regularly receive information or updates about eye health from media, seminars, or health campaigns.	22 (5.90%)	71 (19.10%)	65 (17.50%)	159 (42.90%)	54 (14.60%)
I know the recommended frequency for eye examinations based on my age and health condition.	28 (7.50%)	108 (29.10%)	90 (24.30%)	107 (28.80%)	38 (10.30%)

The most commonly reported barriers to seeking eye care were lack of awareness about available services (73.30%; n = 272), work-related commitments (73.10%; n = 271), and fear of diagnosis or treatment (70.70%; n = 262). Other reported barriers included lack of time (64.70%; n = 240), financial constraints (60.60%; n = 225), and preference for traditional or alternative medicine (58.2%; n = 216). The belief that eye problems will resolve discouraged nearly half of the participants (47.10%; n = 175) from accessing eye care. This is presented in Table 9.

**Table 9:** Barrier of eye care-seeking behavior among staff of University of Benin

Barriers to eye care	Strongly Agree	Agree	Neutral	Disagree	Strongly Disagree
Lack of time prevents me from seeking eye care when needed.	128 (34.50%)	112 (30.20%)	70 (18.90%)	27 (7.30%)	34 (9.10%)
Financial constraints prevent me from seeking eye care when needed.	92 (24.80%)	133 (35.80%)	86 (23.20%)	38 (10.25%)	22 (5.95%)
Fear of diagnosis or treatment prevents me from seeking eye care when needed.	82 (22.20%)	180 (48.50%)	41 (11.10%)	23 (6.10%)	45 (12.10%)
Lack of awareness about available eye care services prevents me from seeking eye care when needed.	115 (31.00%)	157 (42.30%)	53 (14.30%)	6 (1.60%)	40 (10.80%)
Work-related commitments prevent me from seeking eye care when needed.	195 (52.60%)	76 (20.50%)	45 (12.10%)	33 (8.90%)	22 (5.90%)
I prefer traditional or alternative remedies over professional eye care services.	43 (11.60%)	173 (46.60%)	27 (7.30%)	10 (2.70%)	118 (31.80%)
I believe that eye problems will resolve on their own without professional care.	68 (18.30%)	107 (28.80%)	95 (25.60%)	15 (4.10%)	86 (23.20%)

## Discussion

Factors influencing eye care-seeking behavior among staff of the University of Benin

This study examined the association between socio-demographic factors and eye care-seeking behavior, which revealed that gender played a significant role in the determination of utilization patterns. Gender was significantly associated with eye health-seeking behavior (p < 0.05), aligning with findings by Thompson et al., which reported that gender significantly influences healthcare service utilization [18]. Vela et al. further corroborated this, observing that gender influences health-seeking behavior, as women were significantly more likely to undergo recent eye exams (p < 0.01) [6]. Similarly, Neyhouser et al.’s study in Cambodia found that gender likely affects eye care-seeking behavior [19], potentially due to differences in health consciousness and societal norms [20]. Abdul-Kabir et al. highlighted a gender-based trend in their study, noting that female participants constituted a higher proportion of visits to health care facilities for eye health-related problems, though this difference was not statistically significant (p = 0.134) [15]. However, Arinze et al. observed that while gender was a significant (p = 0.017) factor of eye care service utilization in bivariate analyses, it did not remain a significant independent predictor in the multivariate logistic model [14].

In contrast, age, marital status, educational level, and job rank showed no significant association (p > 0.05) with eye care-seeking behavior. While education is often linked to improved health literacy and proactive health-seeking, this study found no such influence, contradicting prior research that identified education and urban residency as key determinants [21]. Similarly, Ogunrinde and Aluko’s finding that age impacts eye health-seeking behavior was not replicated here [22]. Also, socioeconomic status (reflected by job rank) did not predict utilization, unlike the study by Abdul-Kabir et al. [15], suggesting equitable access to eye care services across staff ranks.

Health financing, however, was significantly associated (p < 0.05) with eye care-seeking behavior. Participants with NHIS coverage (56.3%) were more likely to utilize services than those relying on out-of-pocket payments (41.5%) or other funding sources (2.2%). This mirrors Eze and Chukwuma’s conclusion that health insurance reduces financial barriers and Ocansey et al.’s finding that 60.6% of uninsured individuals never sought eye care, compared to 39.5% of insured participants [23,24]. Reliance on out-of-pocket payments remains a critical barrier, as noted by Mohammadi et al. [25]. 

Awareness and knowledge of eye care delivery among staff of the University of Benin

Furthermore, this study also sought to determine the level of awareness of eye care delivery among staff at the University of Benin. The results showed that a majority of the participants (78.40%) acknowledged the importance of regular eye examinations, and 52.50% were familiar with the different types of eye care services, such as optometry and ophthalmology. However, a notable knowledge gap was identified regarding the recommended frequency of eye examinations (39.10%), which may affect preventive care behaviors. This is consistent with a study by Evans et al., which found that even among educated populations, health literacy gaps persist [26].

Although most of the participants were aware of the importance of seeking eye care, access to accurate and timely information on eye health was lacking. Many participants did not receive regular updates on eye health from media, seminars, or health campaigns (57.50%). Similar trends were reported in Saudi Arabia, where Fallatah found that awareness levels did not necessarily translate into active eye care-seeking behavior [27]. This gap suggests a disconnect between awareness and action, reinforcing the need for targeted, workplace-specific awareness campaigns to enhance proactive health-seeking behavior.

Barriers to eye care-seeking behavior among staff of the University of Benin

Another important objective of this study was to assess whether specific barriers were associated with differences in eye care-seeking behavior. The findings showed multiple obstacles, mostly including a lack of awareness about available eye care services (73.30%), work-related commitments (73.10%), and fear of diagnosis or treatment (70.70%). These barriers significantly influenced the ability and willingness of staff to seek timely eye care.

Lack of awareness about available eye care services was the most frequently reported barrier. This suggests that although some staff were aware of the importance of eye care, they did not know where to access appropriate services or the types of care available. Similar findings have been reported by Atuanya and Chilaka, who found that even when individuals knew about available eye care services, it did not always lead to utilization [13]. To ameliorate this gap, there is a need for better communication strategies to be implemented, such as workplace awareness programs, digital information campaigns, and structured university health outreach initiatives.

Work-related commitments emerged as another key barrier, as many university staff members prioritize their job responsibilities over health check-ups, which is consistent with findings from Yao et al. [28]. This study found that pressure from work often impedes healthcare appointments, leading to delayed health-seeking behavior. Introducing periodic vision screenings at work and flexible appointment scheduling could help mitigate this challenge.

Fear of diagnosis or treatment was another major factor, which suggests that anxiety about receiving a diagnosis or undergoing an eye examination discourages many staff members from seeking eye care. Similar concerns were noted in a study by Frempong and Van Staden, which found that the fear of undergoing surgery often prevents individuals from seeking eye care services [29]. Psychological barriers like fear of dependency on glasses and fear of surgery may contribute to delays in treatment, which can lead to preventable vision loss [30]. Addressing this issue requires patient-centered education and counseling services to reduce healthcare-related anxiety. Other barriers reported in this study were a lack of time, which prevented the participants from seeking eye care when needed (64.70%), financial constraints (60.60%), and preference for traditional or alternative medicine (58.2%).

Limitations and recommendations

While this study provided valuable findings into the eye care-seeking behavior among university staff, several limitations must be acknowledged. Multiple replies were invalidated as they were incomplete. In addition, the sample size calculation was based on the 2019/2020 staff population due to the unavailability of updated institutional records at the time of study design; however, actual recruitment occurred during the 2023/2024 academic session, which may introduce minor variability in proportional representation. The authors also recommend that future studies employ more rigorous analyses so as to provide better insights into health care uptake among similar communities.

## Conclusions

This study showed that while awareness of eye care is relatively high among staff at the University of Benin, various barriers prevented the effective utilization of the services, especially a lack of awareness about available eye care services. Also, gender and health financing were significant determinants of eye care-seeking behavior. However, age, marital status, education level, and job rank showed no significant influence on utilization patterns. These findings emphasize the need for targeted interventions, such as workplace eye health programs, awareness campaigns, and improved access to affordable eye care services, to bridge the gap between awareness and healthcare utilization.
